# Rural Older Adults’ Experiences With Pain From Chronic Illnesses and Its Treatment

**DOI:** 10.1093/geroni/igab046.1122

**Published:** 2021-12-17

**Authors:** Hyunjin Noh, Zainab Suntai, Cho Rong Won

**Affiliations:** 1 University of Alabama, Tuscaloosa, Alabama, United States; 2 University of Alabma, Tuscaloosa, Alabama, United States

## Abstract

Although pain control is an essential factor in promoting quality of life, pain is undertreated among certain sub-populations, such as older adults and rural residents. The purpose of this study was to explore pain experiences and its treatment among rural older adults. A qualitative research design was adopted to capture the common essence of participants’ experiences through a phenomenological method. Purposeful sampling was used, and the participant criteria was: age 55+, have good thinking skills, resident of Alabama, have chronic/serious health conditions, and experienced pain or discomfort in the last 3 months. Twenty-three participants were recruited from rural counties of West and South Alabama through the local Area Agency on Aging and health and senior service centers. Individual semi-structured interviews were conducted via phone and were recorded and transcribed verbatim. Thematic analysis was conducted to identify emerging themes and repeated patterns from the data. Our results revealed themes in four categories: 1) impact of pain: physical limitations and coping strategies, 2) Impact of Covid-19: physical health, social, and mental health impact, 3) challenges in pain treatment: transportation (driver/time/cost/Covid-19 exposure) and non-transportation related problems (lacking resources/mistrust/limited health insurance coverage), and 4) suggestions: transportation-related (more transportation options/financial assistance) and non-transportation-related support (improved insurance coverage/non-pharmacological care) . Findings of this study highlight rural older adults’ unique needs in access to pain treatment, further amplified during the Covid-19 pandemic. Increase in sustainable, funded transportation programs and the supply of local pain specialists is critical to meet such needs and improve their quality of life.

